# Cucurbitacin B Induced ATM-Mediated DNA Damage Causes G2/M Cell Cycle Arrest in a ROS-Dependent Manner

**DOI:** 10.1371/journal.pone.0088140

**Published:** 2014-02-04

**Authors:** Jiajie Guo, Guosheng Wu, Jiaolin Bao, Wenhui Hao, Jinjian Lu, Xiuping Chen

**Affiliations:** State Key Laboratory of Quality Research in Chinese Medicine, Institute of Chinese Medical Sciences, University of Macau, Macao, China; Queensland University of Technology, Australia

## Abstract

Cucurbitacins are a class of triterpenoids widely distributed in plant kingdom with potent anti-cancer activities both *in vitro* and *in vivo* by inducing cycle arrest, autophagy, and apoptosis. Cucurbitacin B (Cuc B), could induce S or G2/M cell cycle arrest in cancer cells while the detailed mechanisms remain to be clear. This study was designed to precisely dissect the signaling pathway(s) responsible for Cuc B induced cell cycle arrest in human lung adenocarcinoma epithelial A549 cells. We demonstrated that low concentrations of Cuc B dramatically induced G2/M phase arrest in A549 cells. Cuc B treatment caused DNA double-strand breaks (DSBs) without affecting the signal transducer and activator of transcription 3 (STAT3), the potential molecular target for Cuc B. Cuc B triggers ATM-activated Chk1-Cdc25C-Cdk1, which could be reversed by both ATM siRNA and Chk1 siRNA. Cuc B also triggers ATM-activated p53-14-3-3-σ pathways, which could be reversed by ATM siRNA. Cuc B treatment also led to increased intracellular reactive oxygen species (ROS) formation, which was inhibited by N-acetyl-l-cysteine (NAC) pretreatment. Furthermore, NAC pretreatment inhibited Cuc B induced DNA damage and G2/M phase arrest. Taken together, these results suggested that Cuc B induces DNA damage in A549 cells mediated by increasing intracellular ROS formation, which lead to G2/M cell phase arrest through ATM-activated Chk1-Cdc25C-Cdk1 and p53-14-3-3-σ parallel branches. These observations provide novel mechanisms and potential targets for better understanding of the anti-cancer mechanisms of cucurbitacins.

## Introduction

**Figure 1:**
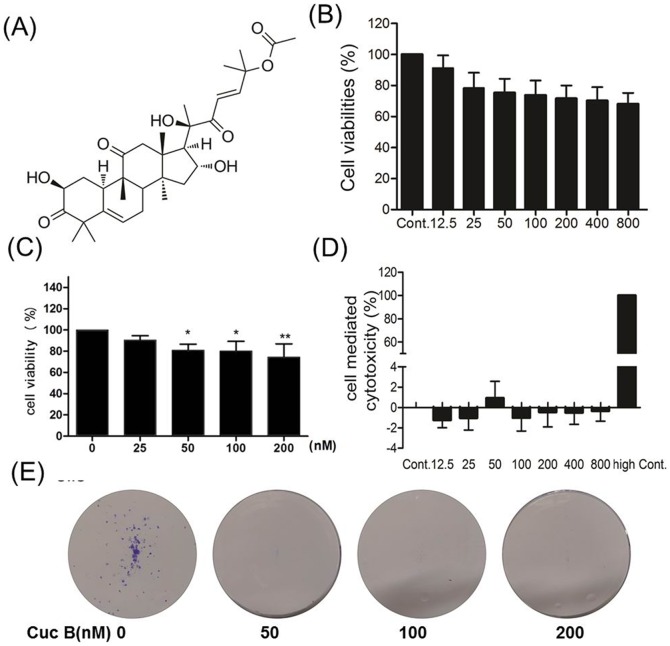
The structure of Cuc B (A). Low concentrations of Cuc B does not significant inhibit cell proliferation after 24 h treatment (B) but prolonged treatment (72 h) inhibit cell proliferation in A549 cells (C). Low concentrations of Cuc B does not affect LDH release in A549 cells after 24 h treatment (D). Low concentrations of Cuc B dramatically inhibit colony formation in A549 cells (E). Values are means ± S.E.M of three independent experiments with five replicates, each conducted in triplicate. Cont, control group.

Cucurbitacins, a class of highly oxidized tetracyclic triterpenoids, are widely distributed in the plant kingdom. To date, more than one hundred cucurbitacins and their derivatives have been identified while only a few of them have been widely investigated. Naturally, cucurbitacin B (Cuc B, Fig 1A) and D are the most common and have the highest content in many plants, followed by E, G, H, and I. Documented data demonstrated that cucurbitacins possess some pharmacological activities, such as anti-inflammation, hepatoprotection, and among others [1], [2].

In the past ten years, the anti-cancer effect of cucurbitacins has drawn attention of many researchers. Recent advances showed that cucurbitacins are potent anti-cancer natural products in both *in vitro* and *in vivo* models. Cucurbitacins dramatically inhibit the growth and proliferation of a series of cancer cells. They could also induce cancer cell differentiation, inhibit angiogenesis and metastasis [2], [3]. Previous studies showed that cucurbitacins significantly inhibited cell growth by interfering with actin dynamics [4]–[7]. Furthermore, cucurbitacins have been identified as small molecular inhibitors of signal transduction and activator of transcription-3 (STAT3) [8]–[10]. Therefore, F-actin and STAT3 have been generally considered as their potential molecular targets in cancer cells.

Accumulated data showed that cucurbitacins could induce different phases of cell cycle arrest depending on the type of cucurbitacins and the type of cell line. It has been reported that Cuc B induced S-phase arrest in BEL-7402, HL60, and U937 cells as well as G2/M-phase arrest in Panc-1, MiaPaCa-2, K562, SW480, and Hep-2 cells. Cuc E and I caused G2/M phase arrest in Panc-1, BEL-7402, HepG2, HL60, T24, and ES-2 cells while Cuc D led to S phase arrest in myeloid leukemia cells [2]. In pancreatic cancer cell lines, Cuc B-induced G2/M phase arrest might be mediated by inhibiting activated JAK2, STAT3, and STAT5, increasing level of p21(WAF1), and decreasing expression of cyclin A, cyclin B1 [11]. While in BEL-7402 human hepatocellular carcinoma cells, Cuc B induced S-phase arrest was considered to be due to its inhibition of cyclin D1 and Cdk1 expression but without affecting STAT3 phosphorylation [12]. However, the detailed underlying mechanisms remain to be clear.

Intracellular reactive oxygen species (ROS) has been implicated in a wide range of biological activities and disease states such as atherosclerosis, diabetes, cancer, neurodegeneration, and aging [13]. Cuc B induced intracellular ROS formation in SW480 cells, which played an important role in G2 cycle arrest and apoptosis [14]. Cuc B induced mitochondrial ROS production also contributed to autophagy in HeLa cells [15]. Excess ROS production could cause different kinds cell damage, including the oxidative injury of DNA [16], which can *via* checkpoint activation induce cell cycle arrest [17]. In the DNA damage response, activation of DNA damage checkpoints is firstly recognized by sensors proteins, followed by activation of a series effector kinases which target the major cell cycle control machinery [18]. ATM (Ataxia telangiectasia mutated) and ATR (Ataxia telangiectasia and Rad3 related), two important transducer proteins, play critical roles in DNA damage response by controlling the damage response through phosphorylation of effector proteins [19], [20]. Following their activation and phosphorylation, the downstream effectors such as Chk1, Chk2, p53 were phosphorylated and activated, leading to further transmission of the checkpoint signals [21], [22]. By the interaction between the cyclin-dependent kinases (Cdks) and the cyclins, cells transit between different phases of cycle. CDKs was activated by dephosphorylation on Thr-14 and Tyr-15 through Cdc25, which complete phases transition [23]–[25]. Cdc25 can be phosphorylated by Chk1/Chk2 on Ser-323 (Cdc25B) and Ser-216 (Cdc25C) and functionally inactivated by binding to 14-3-3 proteins [26]–[28].

In the present study, we examined the effect and potential mechanisms of Cuc B on cell phases in A549 cells. We demonstrated that Cuc B causes G2/M phase cell cycle arrest in A549 cells, which is associated with DNA damage mediated by ATM-activated Chk1-Cdc25C-Cdk1 and p53-14-3-3-σ parallel branches. The DNA damage was mediated by the accumulation of intracellular ROS formation. These findings dissect the role of ROS and DNA damage in Cuc B induced G2/M arrest, and might offer some potential therapeutic targets for this natural product.

## Materials and Methods

### Chemicals and antibodies

Cucurbitacin B, purchased from ShunBo Biological Engineering Technology Co., Ltd (Shanghai China), was dissolved in dimethyl sulfoxide (DMSO) to make 200 µM stock solutions and was kept at −20°C. The stock solutions were freshly diluted to the desired concentration just before use. N-acetyl-L-cysteine (NAC) was purchased from Beyotime (Haimen, China). Specific antibodies against GAPDH, phospho-Cdc25C-Ser-216, Cdc25C, phospho-p53-Ser-15, phospho-p53-Ser-20, p53, phospho-STAT3-Tyr-705, STAT3, phospho-ATM-Ser-1981, phosphor-ATR-Ser-428, ATR, phospho-Chk1-Ser-345, Chk1, phosphor-Chk2-Thr-68, Chk2 were purchased from Cell Signaling Technology (USA). Phospho-Cdk1-Tyr-15, Cyclin B1, 14-3-3-σ were from Santa Cruz Biotechnology (USA). Cdk1 antibody was obtained from BD Transduction Laboratories™ (USA). Antibodies for ATM and γH2AX were obtained from GeneTex and Millipore respectively.

### Cell culture

Adenocarcinomic human alveolar basal epithelial cells (A549 cells) and human breast cancer cells (MCF-7 cells) (ATCC) were cultured in RPMI 1640 medium supplemented with 10% fetal bovine serum, 100 U/mL penicillin and 100 µg/mL streptomycin (GIBCO, USA), and were grown in an incubator with 5% CO_2_ at 37°C.

### XTT assay and LDH release assay

Exponentially growing A549 cells were planted into 96-well plates and were treated with a series of concentrations of Cuc B after adhesion. The cell viability was determined after 24 h-incubation by adding 50 µl XTT mixture solution (Roche, Germany). After 4 h-incubation, the XTT-containing medium was detected using a Multilabel counter (Perkin Elmer, Singapore) by measuring the absorbance at 450 nm with a reference wavelength at 650 nm. The cell viability was determined after 72 h-incubation was also determined.

The cells were cultured and treated as mentioned above. The LDH released to the culture medium was detected with a commercial LDH assay kit (Roche, Germany) followed by manufacturer's instructions.

### Colony formation assay

A549 cells were seeded into 6-well plates at a density of 200 cells per well and treated with different concentrations of Cuc B. After one weeks, cells were fixed using 4% paraformaldehyde and stained with Crystal Violet Staining Solution (Beyotime Institute of Biotechnology, China). The visible colonies (≥50 cells) were photographed by a common NIKON camera.

### Comet assay

The DNA damage was evaluated using the comet assay as previously described with minor modifications [29]. Briefly, Cuc B treated cells were harvested and mixed with 0.75% low melting point agarose and layered onto microscope slides pre-coated with 0.75% normal melting point agarose. Then the slides were submerged in pre-chilled lysis solution (1% Triton X-100, 2.5 M NaCl, 1% laurosylsarcosinate and 10 mM EDTA, pH 10.5) for 1 h at 4°C. After soaking with pre-chilled unwinding and electrophoresis buffer (0.3 M NaOH and 1 mM EDTA, pH 13) for 20 min, the slides were subjected to electrophoresis for 15 min at 0.5 V/cm (20 mA), and then stained with PI. Individual cells were viewed using an Olympus IX81 fluorescence microscope.

### Cell cycle distribution and phase determination

After Cuc B incubation, cells and transfected cells were washed with PBS and fixed with 70% ethanol solution overnight at −20°C. After PBS washes the cells were stained with 5 µg/ml RNase and 20 µg/ml PI (propidium iodide/RNase A staining buffer, Invitrogen) in the dark at 37°C for 30 min and then analyzed with a flow cytometry (Becton Dickinson FACS Canto™, Franklin Lakes, NJ). At least 10,000 events were counted for each sample. The DNA content in the G0/G1, S, and G2/M phases were analyzed using ModFit 161 LT version 3.0 software (Verity Software House, Topsham, USA).

### Western blotting assay

After Cuc B treatment, the protein expressions in cells and transfected cells were determined by Western blotting. Briefly, after quantitative determination of protein content in each sample by BCA™ Protein Assay Kit (Pierce), 40 µg proteins were subjected to 6–12% SDS-PAGE and transferred onto Immun-Blot PVDF Membrane (Bio-Rad Laboratories). After blocking with 5% non-fat milk in TBST (20 mM Tris-HCl, 500 mM NaCl, and 0.1% Tween 20) at room temperature for 1 h, the membranes were incubated with specific primary antibodies for overnight at 4°C. After washing with 5% non-fat milk/TBST, the membranes were incubated with horseradish-peroxidase-conjugated secondary antibodies (Santa Cruz Biotechnology) at room temperature for 1 h. The protein-antibody complexes were detected by ECL Advanced Western Blot detection Kit.

### Cell transfection with siRNA

Briefly, approximate 1.5×10^5^/well cells were seeded in 6-well plate for overnight. For per well, diluted 100 pM siRNA in 100 µl Opti-MEM reduced serum medium and mixed gently. Diluted 5 µl lipofectamine™ 2000 (Invitrogen™) in 100 µl of Opti-MEM reduced serum medium, and mixed gently. The mixtures were incubated for 5 min at room temperature. Then the diluted siRNA and the diluted lipofectamine (total volume 200 µl) were mixed gently and incubate for 20 min at room temperature. 200 µl of siRNA-lipofectamine complexes was added to each well containing cells and 800 µl Opti-MEM reduced serum medium. After 12 h incubation, the complexes were removed and cells were cultured with completed medium. After incubation 6 h, cells were treated with Cuc B for further experiments. The siRNA sequences were listed as following: siRNA sequences for ATM, 5′-GGGCAAUAUUUCAAAUUAATT-3′, 5′-UUAAUUUGAAAUAUUGCCCTT-3′; siRNA sequences for Chk1, 5′-GCGUGCCGUAGACUGUCCATT-3′, 5′-UGGACAGUCUACGGCACGCTT-3′; Negative control sequences, 5′-UUCUCCGAACGUGUCACGUTT-3′, 5′-ACGUGACACGUUCGGAGAATT-3′.

### Immunoprecipitation (IP) assay

Approximate 10^6^ cells were plated and treated with/without ATM siRNA and Cuc B for 24 h. Cells were washed twice with ice-cold PBS and were lysed on ice with Beyotime™ lysis buffer. The cell lysate was collected by centrifugation at 3,000 g for 15 min. Approximate 10 µg of 14-3-3-σ antibody was added to 300 µg of lysate obtained from cells and incubated overnight at 4°C. Agarose A (Santa Cruz Biotechnology) beads (20 µl) were added to the mixture and were incubated for another 8 h with gentle rocking. Lysates were then collected by centrifuging at 3000 g for 3 min and supernatant was separated. Beads were then washed 6 times with lysis buffer. 14-3-3-σ complex from each sample was eluted with 40 µl of lysis buffer. Then, the protocol follows Western blotting.

### Measurement of intracellular ROS production

After cells were treated with Cuc B, 5-(6)-carboxy-2′,7′-dichlorodihydrofluorescein diacetate (DCFH_2_-DA; Molecular Probes) was added (final concentration 20 µM) and incubated in the dark at 37°C for 30 min. Cellular fluorescence for a sample of 10^4^ cells was analyzed using a flow cytometry (Becton Dickinson FACS Canto™, Franklin Lakes, NJ) with wavelength of excitation and emission at 488 nm and 525 nm respectively.

### Statistical analysis

Data were expressed as the means ± SEM from at least three separate experiments performed triplicate. The differences between groups were analyzed using Prism 5.0 (GraphPad Software Inc., San Diego, CA) and *p*<0.05 is considered statistically significant.

## Results

### Cuc B inhibited A549 cells proliferation and colony formation

Cuc B has been shown to inhibit multiple cancer line cells proliferation in a time- and dose- dependent manner [12], [30], [31]. In present study, we also observed that Cuc B significantly inhibit A549 cell proliferation in a dose- and time- dependent manner (Fig. S1). However, low Cuc B concentration (<800 nM) showed no obvious effect on cell viability after 24 h treatment (Fig. 1B). To confirm this, the LDH release was determined. Result showed that no obvious LDH release in the culture medium was detected even after 800 nM Cuc B treatment for 24 h (Fig. 1D). However, prolonged treatment with Cuc B for 72 h, it dose-dependently inhibited A549 proliferation (Fig. 1C). Furthermore, the colony formation assay *in vitro* demonstrated that the colony formation was dramatically suppressed by Cuc B in a dose-dependent manner (Fig. 1E).

### Cuc B induced DNA double-strand breaks (DSBs)

**Figure 2:**
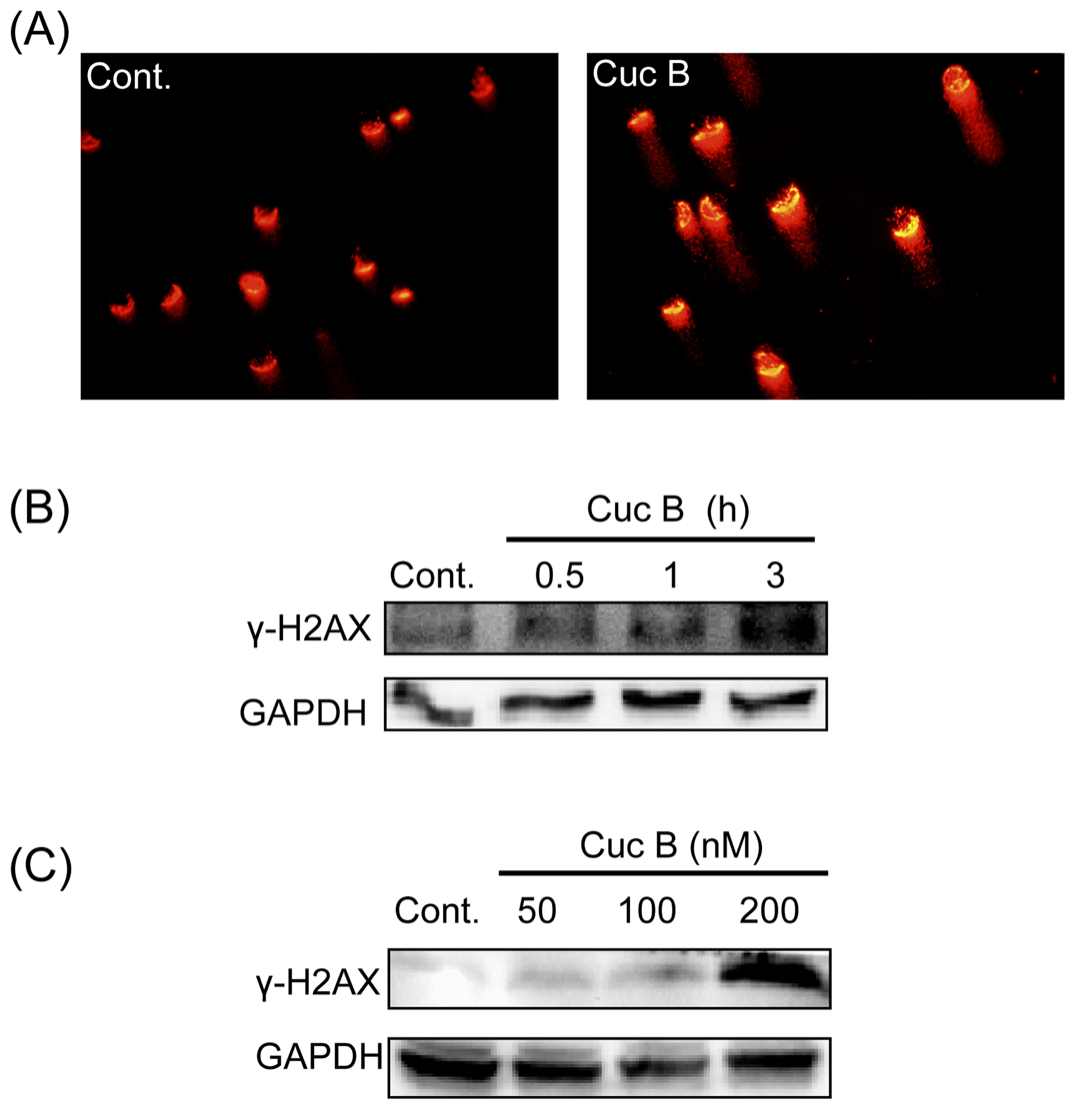
Cuc B induces DNA damage in A549 human lung cancer cells. Cells were treated with 200 nM Cuc B for the 3 h and DNA damage was detected by comet assay. Nuclei with damaged DNA have a comet feature with a bright head and a tail, whereas nuclei with undamaged DNA appear round with no tail. Typical micrographs of comet assays were shown (A). Cells were treated with 200 nM Cuc B for 0.5, 1, 3 h and the level of γH2AX was detected using Western blot analysis (B). Cells were treated with 50, 100, 200 nM Cuc B for 24 h and the level of γH2AX was detected using Western blot analysis (C).

Phosphorylation of the histone variant H2AX on Ser-139 (γH2AX) is an early cellular response to the induction of DNA DSBs, forming discrete nuclear foci at the damaged sites [32], [33]. To test if Cuc B induced DSBs, we examined the protein expression of γH2AX. Cuc B induced γH2AX protein expression in a time- and dose- dependent manner (Fig. 2B, 2C). It was significantly up-regulated as early as 3 h after Cuc B treatment and lasted for 24 h. To confirm the effect of Cuc B on DNA damage in A549 cells, a comet assay (single cell electrophoresis) was performed. The comet tails were significantly longer in Cuc B treated cells than that of control cells after 3 h incubation, indicating that Cuc B induced A549 DNA damage (Fig. 2A). Furthermore, increased protein expression of γH2AX and long comet tails were also observed in a dose-dependent manner in Cuc B treated MCF-7 breast cancer cells (Fig. S2). These results clearly indicated that Cuc B exposure induced DNA damage in both A549 cells and MCF-7 cells.

### Cuc B induced G2/M cell cycle arrest in A549 cells

**Figure 3:**
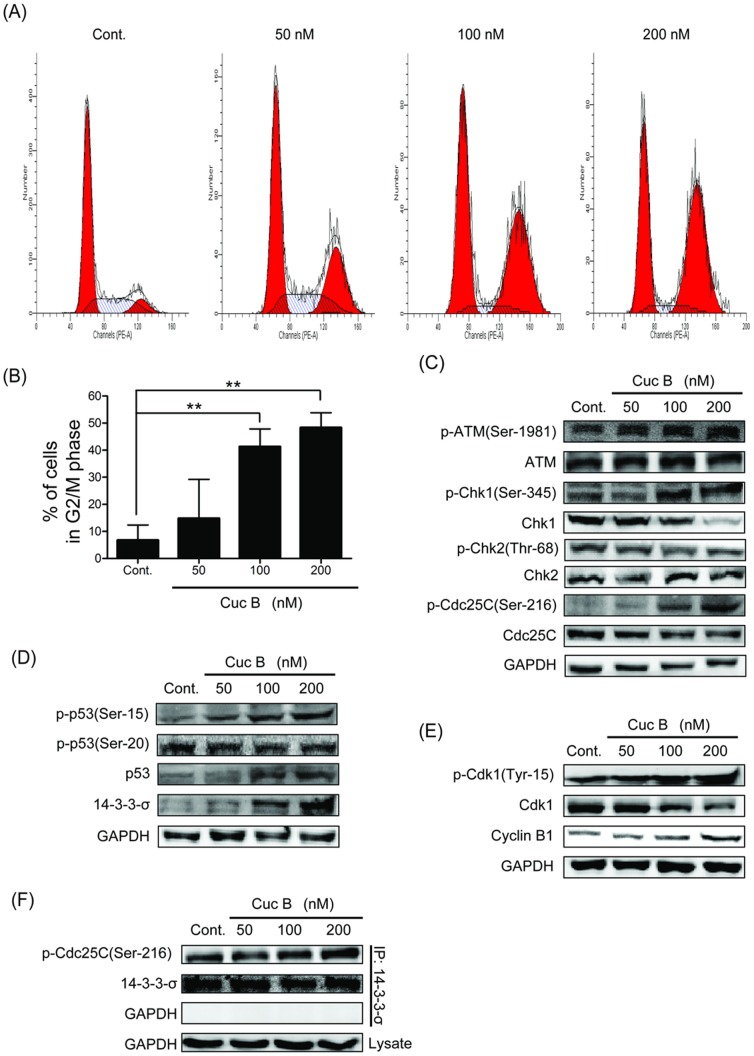
Cuc B induces G2/M phase arrest and modulates cell cycle regulatory proteins in human lung cancer A549 cells. Cell cycle analysis was performed by flow cytometry. Representative cell cycle profiles of A549 cells treated with 50, 100, 200-A represents the intensity of propidium iodide, and the y axis represents the cell counts (A). Concentration-dependent effect of Cuc B was evaluated by flow cytometer to quantitate the number of cells in G2/M phase in A549 cells. Values are means ± S.E.M of three independent experiments, each conducted in triplicate (B). Western blot analyses of ATM, Chk1, Chk2, Cdc25C, p53, 14-3-3-σ, Cdk1, Cyclin B1 and their phophorylated forms in Cuc B exposed A549 cells for 24 h (C-E). Total 14-3-3-σ protein was immunoprecipitated from control and Cuc B treated A549 cells lysates and analyzed for p-Cdc25C (Ser-216) for 24 h (F). ^**^ *p*<0.01 *vs.* Cont. Cont, control group.

Cucurbitacins have been shown to induce cell cycle arrest in S or G2/M phase in a number of cancer line cells. For Cuc B, a number of studies reported that it could induce G2/M phase arrest in Hep-2, MCF-7, K562 cells and S-phase arrest in BEL-7402, HL60, and U937 cells [2]. In this study, we tested the effect of Cuc B on cell cycle. The cell cycle distribution analysis revealed that Cuc B treatment caused significant accumulation of cells in G2/M phase in A549 cells in a dose-dependent manner (Fig. 3A, 3B). In 200 nM Cuc B treated cells, more than half were arrested in G2/M phase (Fig. 3B).

### Cuc B activated ATM-Chk1-Cdc25C-Cdk1 cascade

To elucidate the molecular mechanism leading to Cuc B-mediated G2/M phase arrest, the signaling pathway responsible for G2/M checkpoint control was detected. As cellular responses to DNA damage are coordinated primarily by two distinct kinase signaling cascades, the ATM-Chk2 and ATR-Chk1 pathways [34], we firstly investigated the effect of Cuc B on expression and phosphorylation of ATM and ATR. Compared with control, the phosphorylation of ATM on Ser-1981 was markedly increased after Cuc B treatment while ATM remains unaffected (Fig. 3C). However, no effect of Cuc B on ATR expression and phosphorylation was observed (data not shown). To ascertain the checkpoint-transducer kinases, phosphorylated downstream effectors of ATM/ATR, the phospho-Chk1-S345 and phospho-Chk2-T68 kinases were determined. The phosphorylation of Chk1 at S345 was up-regulated by Cuc B (Fig. 3C) without affecting phosphorylation Chk2 at Thr-68 (Fig. 3C). This result indicated that Chk1 but not Chk2 might play a dominant role in the response to Cuc B induced DNA DSBs. Chk1 activation has been shown to phosphorylate Cdc25C on serine-216 *in vitro* [35]. We further test the effect of Cuc B on phosphorylation of Cdc25C at Ser-216. The level of Ser-216-phosphorylated Cdc25C was dramatically increased in Cuc B treated cells (Fig. 3C) suggesting that activation of Chk1 by Cuc B was associated with inactivation of Cdc25C. Cdc25C is an upstream regulator of Cyclin-B1-Cdk1 [36]. Consistent with increased Cdc25C phosphorylation on Ser-216, the inactive form of Cdk1 (phosphorylation on Tyr-15) was also up-regulated by Cuc B (Fig. 3E). Expression of Cdk1 and Cyclin B1 was down- and up- regulated respectively (Fig. 3E). These results indicated that ATM-Chk1-Cdc25C-Cdk1 signal participated in the G2/M checkpoint in Cuc B induced DNA damage.

### Cuc B activated p53 and enhanced the binding of phosphorylation Cdc25C on Ser-216 and 14-3-3-σ

In response to DNA damage, enhanced phosphorylation of p53 on Ser-15 by ATM has been reported [37]. p53 could also be activated by Chk1/Chk2 at Ser-20. Thus, the effect of Cuc B on p53 phosphorylation was studied. Cuc B induced phosphorylation of p53 on Ser-15 without affecting phosphorylation of Ser-20. Interestingly, expression of p53 was also significantly up-regulated in the meantime (Fig. 3D). 14-3-3-σ is a p53-regulated downstream inhibitor of G2/M progression [38]. Following p53 activation by Cuc B, the expression of 14-3-3-σ was significantly increased (Fig. 3D). In G2 checkpoint control, Ser-216 phosphorylation and 14-3-3 binding negatively regulates Cdc25C [35]. Immunoprecipitation assay showed that the binding of phosphorylation Cdc25C on Ser-216 and 14-3-3-σ was increased (Fig. 3F). These results suggested that ATM-p53-14-3-3-σ participated in the G2/M checkpoint in Cuc B induced DNA damage.

### ATM knockdown reversed Cuc B induced G2/M Arrest

**Figure 4:**
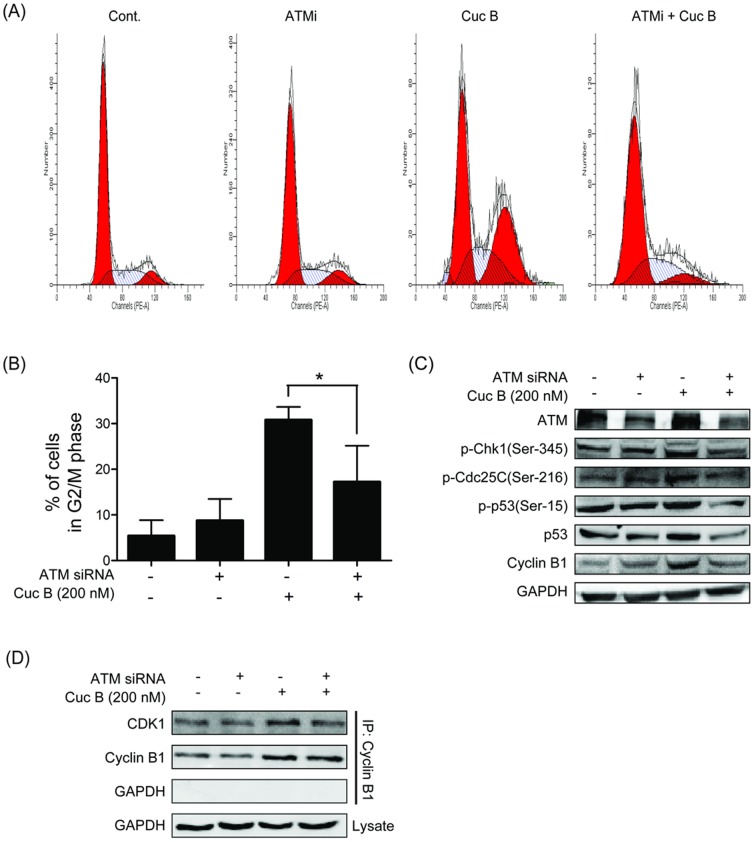
ATM knocked down reversed Cuc B induced G2/M phase arrest. A549 cells transfected with 100(A, B). A549 cells transfected with ATM siRNA and treated with or without 200 nM Cuc B for 24 h. Phosphorylation of Chk1 on Ser-345, Cdc25C on Ser-216, p53 on Ser-15 and protein levels of p53 Cyclin B1 were analyzed by Western blot (C). Total Cyclin B1 protein was immunoprecipitated from ATM siRNA transfected and treated with or without 200 nM Cuc B A549 cells lysates and analyzed for Cdk1 for 24 h (D). ^*^ *p*<0.05 *vs.* Cont. Cont, control group.

To further establish the role of ATM in Cuc B-mediated G2/M phase arrest, transiently transfect A549 cells with ATM siRNA was performed. ATM siRNA transfection dramatically reversed Cuc B induced ATM activation (Fig. 4C) and G2/M phase arrest (Fig. 4A, 4B). The ATM activated Chk1-Cdc25C-Cdk1 pathway was further investigated. Cuc B induced phosphorylation of Chk1 on Ser-345, phosphorylation of Cdc25C on Ser-216, and phosphorylation p53 on Ser-15 were all inhibited by ATM knockdown (Fig. 4C). Similarly, Cuc mediated ATM downstream effector of p53, 14-3-3-σ expression is down-regulated by ATM siRNA. Furthermore, Cuc B up-regulated Cyclin B1 was also reversed by ATM siRNA (Fig. 4C). To test the effect of ATM siRNA on Cuc B induced Cdk1 and Cyclin B1 interactions, IP was performed. Compared with Cuc B treated group, a dramatic decrease of Cyclin B 1-bound Cdk1 was observed in ATM knockdown and Cuc B co-treatment (Fig. 4D).

### Chk1 knockdown reversed Cuc B induced G2/M phase arrest

**Figure 5:**
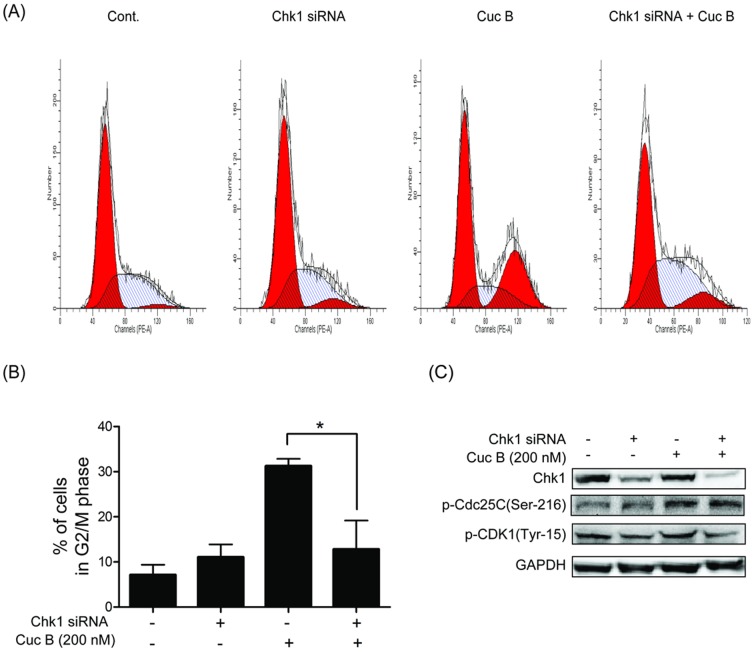
Chk1 knockdown reversed Cuc B induced G2/M phase arrest. A549 cells transfected with 100(A, B). A549 cells transfected with Chk1 siRNA and treated with or without 200 nM Cuc B for 24 h. Phosphorylation of Cdc25C on Ser-216, Cdk1 on were analyzed by Western blot (C). ^*^ *p*<0.05 *vs.* Cont. Cont, control group.

To dissect the downstream effector in Cuc B mediated G2/M phase arrest, the role of Chk1 was examined with Chk1 siRNA. Similar to that of ATM siRNA, Cuc B- induced G2/M arrest in A549 cells was significantly decreased by Chk1 siRNA treatment (Fig. 5A, 5B). Furthermore, Cuc B caused phosphorylation of the Chk1 downstream effector Cdc25C on Ser-216 and Cdk1 on Tyr-15 were also inhibited (Fig. 5C).

### Cuc B induced ROS generation and did not affect STAT3 phosphorylation

**Figure 6:**
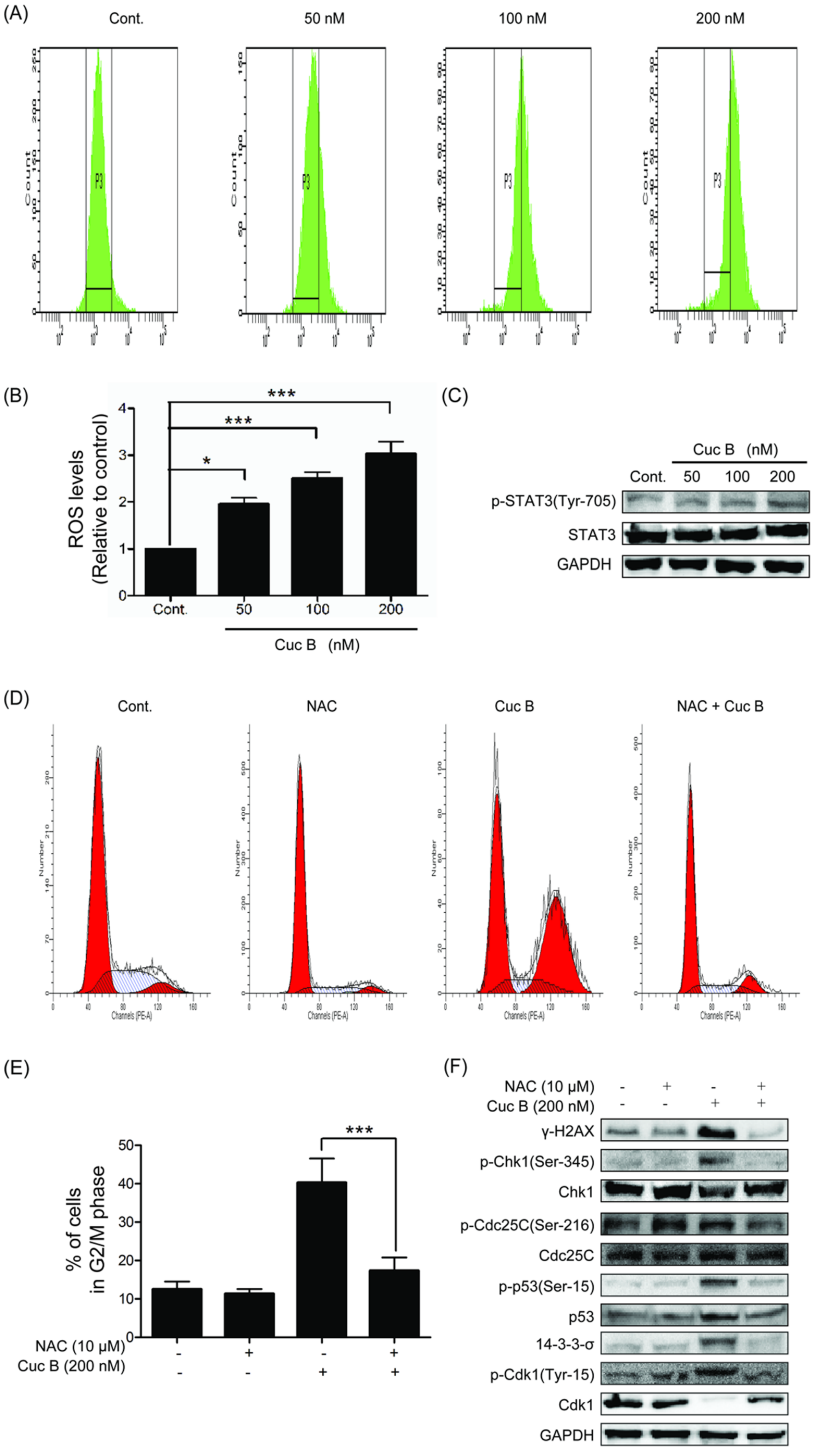
Cuc B induced DNA DSBs active G2/M checkpoint mediated by ROS generation. The generation of ROS in A549 cells after 50, 100, 200_2_-DA as described under Materials and Methods (A, B). Effect of Cuc B on STAT3 phosphorylation on Tyr-705 and STAT3 expression were analyzed by Western blot assay (C). A549 cells were treated with 10 mM NAC for 0.5 h followed by treatment with 200 nM Cuc B for 24 h, and the cell cycle was tested (D, E). A549 cells pretreated with 10 mM NAC for 0.5 h and treated with or without 200 nM Cuc B for 24 h. Phosphorylation of Chk1 on Ser-345, Cdc25C on Ser-216, p53 on Ser-15 and protein levels of Chk1, Cdc25C, p53, 14-3-3-σ, Cdk1 were analyzed by Western blot assay (F). ^*^ *p*<0.05 *vs.* Cont, ^***^ *p*<0.001 *vs.* Cont. Cont, control group.

Recent studies have shown that Cuc B induced intracellular ROS formation in HeLa, SW480, and B16F10 cells [14], [15], [39]. We investigated whether Cuc B induced ROS production in A549 cells. Cuc B significantly induced ROS formation in a dose dependent manner in A549 cell (Fig. 6A, B) which could be inhibited by NAC pretreatment (data not shown). Cucurbitacins has been identified as effective small molecular inhibitor of STAT3 and could dramatically suppress STAT3 phosphorylation [8], [11]. To test whether STAT3 play a role under our experimental conditions, the expression of STAT3 was investigated. We found that Cuc B (200 nM) did not affect the expression of STAT3, nor it affect the phosphorylation of STAT3 at Tyr-705 (Fig. 6C). Thus, these data suggested that Cuc B induced ROS generation in A549 cells but showed no effect on STAT3 under our experimental conditions.

### Cuc B induced G2/M arrest and DNA DSBs was mediated by ROS

Cuc B induced ROS formation has been shown to mediate autophagy [15], depletion of the G-actin pool [39] and G2 phase arrest and apoptosis [14]. Here, we test the role of ROS in G2/M arrest and DNA DSBs in A549 cells. NAC, a general antioxidant and a precursor of glutathione, could almost completely reverse Cuc B induced G2/M phase arrest (Fig. 6D, E). NAC pretreatment was able to block the phosphorylation of γH2AX, suggesting the involvement of ROS in Cuc B-mediated DNA damage (Fig. 6F). We further determined the effect of NAC on Cuc B induced phosphorylation level of G2/M regulatory proteins such as Chk1 on Ser-345, Cdc25C on Ser-216, Cdk1 on Tyr-15, p53 on Ser-15, and the expression of 14-3-3-σ. Results showed that Cuc B induced up-regulation and phosphorylation of these proteins were all significantly reversed by NAC pretreatment (Fig. 6F). These data demonstrated that Cuc B causes DNA damage and G2/M checkpoint mediated by ROS generation in ATM-Chk1-Cdc25C-Cdk1 cascade.

## Discussion

More attention has been paid to the anti-cancer effect of cucurbitacins in recent years. Inducing cell cycle arrest by cucurbitacins has been well established while the detailed mechanisms and pathways are largely to be clear. Cuc B, one of the widely investigated cucurbitacins, cause different phase cell cycle arrest in different cancer cells. Previous data suggested that Cuc B caused cell cycle arrest by blocking the STAT3 signaling pathway, which resulted in reduced expression of downstream targets, such as Cyclin B1, Cyclin A [40]–[42]. In SW480 cells, Cuc B induced G2 arrest and apoptosis through a STAT3-independent but ROS-dependent mechanism [14]. In this study, we showed that Cuc B induced G2/M arrest in a ROS dependent manner without affecting STAT3 in A549 cells: Cuc B induced ROS-mediated DNA damage, which activated G2/M phase checkpoint through ATM-activated Chk1-Cdc25C-Cdk1 and -p53-14-3-3-σ cascades.

The anti-proliferative effect of Cuc B on cancer cells has been reported everywhere. Similar to its effect on other reported cancer cells, Cuc B could significantly inhibit A549 cells proliferation and growth in a dose- and time- dependent manner. Though low concentrations of Cuc B showed no significant effect on A549 cell proliferation after 24 h treatment, prolonged treatment significantly inhibited cancer cells proliferation and colony formation clearly demonstrating that Cuc B is a potent cytotoxic compound. It could exert cytotoxicity at very low concentrations (50–200 nM).

STAT3, one of the seven members of the STAT transcription factor protein family, has been implicated as a potential target for cancer therapy. Activation of STAT3 signaling could up-regulate Cyclin B1, c-Myc, Bcl-x and regulating cell growth and survival. STAT3 inhibition has been found to induce cell cycle arrest and apoptosis in STAT3-positive tumor cells [43]–[45]. Furthermore, STAT3 has been identified as one of the important molecular target for cucurbitacins. In this study, we found that low concentrations of Cuc B did not affect STAT3 phosphorylation in A549 cells. Thus, proposing that Cuc B induced G2/M phase arrest in a STAT3 independent manner.

Many chemotherapeutic agents have been shown to induce DNA damage response in cancer cells. The effect of cucurbitacins on DNA damage remains to be clear. In present study, we firstly found that Cuc B induced DNA damage in A549 cells in a dose- and time- dependent manner as evidenced by the long tails in comet assay, which was further confirmed by the upregulated expression of γH2AX, a biomarker/sensor for DNA damage. Similar results were also observed in Cuc B treated MCF-7 cells. These results suggested that DNA damage induction might be a common effect of Cuc B. PI3 Kinase-related kinase (PIKK) family members, ATM and ATR are the key protein kinases response DNA damage to activate checkpoints. Response to DNA damage, ATM and ATR are the firstly activated by autophosphorylation [19], [46], [47]. In present study, ATR was not affected after Cuc B exposure, but enhanced phosphorylation of ATM on Ser-1981 and phosphorylation histone H2AX on Ser-139 was observed suggesting the involvement of ATM. The DNA damage was observed as early as 3 h after Cuc B treatment suggesting that it is an early event after Cuc B incubation. Generally, ATM activates Chk2 by phosphorylation it on Thr-68 [48], [49], while ATR activates Chk1 [50]. However, Chk1 could be activated by both ATR and ATM on Ser-345 [51]. In this study, a significant increase of phosphorylation of Chk1 on Ser-345 after Cuc B exposure was observed, whereas the phosphorylation of Chk2 on Thr-68 was not affected. To establish the role of ATM in Cuc B-mediated G2/M phase arrest in A549, ATM was knocked down by transfection with ATM siRNA. Cuc B-mediated G2/M phase arrest was dramatically reversed by ATM siRNA transfection. Cuc B caused Chk1 phosphorylation is also blocked by ATM siRNA. Similarly, Chk1 knocked down reversed Cuc B induced G2/M phase arrest. Thus, these results illustrated that Cuc B induced G2/M phase arrest in A549 cells *via* ATM-Chk1 pathway.

ATM-activated Chk1 can phosphorylate Cdc25C, contributing to G2/M phase checkpoints [52]. Cdc25C is essential for promoting mitosis though dephosphorylating Tyr-15 on Cdk1 [53]. Phosphorylation of Cdc25C on Ser-216 is an inactive state of Cdc25C, which made a binding site for proteins of the 14-3-3-σ. The binding of phosphorylated Cdc25C with 14-3-3-σ in the cytoplasm prevents Cdc25C from dephosphorylating the cycling-dependent kinase Cdk1, resulting in cells arrest in G2/M phase [28], [35], [54]. Our results showed that Cuc B induced phosphorylation Cdc25C on Ser-216 in a dose-dependent manner, which could be blocked by ATM siRNA and Chk1 siRNA suggesting that Cdc25C was another downstream effector in Cuc B induced DNA damage response. Additionally, DNA damage could induce ATM to activate p53 through phosphorylating it directly on Ser-15 and/or on Ser-20 *via* Chk1/Chk2 [55]. We found that Cuc B exposure induced p53 phosphorylation on Ser-15 but not on Ser-20 illustrating that ATM directly activated p53 by phosphorylation on Ser-15. This contributes primarily to enhance the activity of p53 as a transcription factor. The 14-3-3-σ, a gene directly regulated by p53 [54], is induced by DNA damage and is required for G2/M phase arrest. Our results showed that the expression of 14-3-3-σ was increased after Cuc B treatment. Furthermore, the increased p53 phosphorylation on Ser-15 and 14-3-3-σ expression by Cuc B were reversed by ATM siRNA. In addition, the binding of 14-3-3-σ with Cdc25C phosphorylation on Ser-216 increased after Cuc B treatment. Thus, an ATM-p53-14-3-3-σ branch pathway might exist in Cuc B induced DNA damage response. When Cdc25C is in inactive status, Cdk1 keeps an inhibitory phosphorylation on Tyr-15. Cell phase progression from G2 to M phase is highly dependent upon the activity of the Cyclin B/Cdk1 complex which is inactivated *via* inhibitory phosphorylation of conserved Thr-14 and Tyr-15 residues of Cdk1 [23], [25]. We detected the effect of Cuc B on the phosphorylation of Cdk1 on Tyr-15. Cuc B dose-dependently increased phosphorylation of Cdk1 on Tyr-15, which could be inhibited by ATM siRNA and Chk1 siRNA. Furthermore, Cuc B induced G2/M phase arrest in A549 cells could be significantly reversed by ATM siRNA and Chk1 siRNA. All the results showed that Cuc B induced DNA damage and G2/M checkpoint in A549 cells through ATM activated Chk1-Cdc25C-Cdk1 and p53-14-3-3-σ pathways.

Previous studies reported that Cuc B induced high level of intracellular ROS formation contributing to its effect on cell cycle arrest, apoptosis and autophagy [14], [56]. ROS induced oxidative DNA damage has been well established. In this study, we found that Cuc B induced high level of ROS formation in A549 cells, which could be almost completely suppressed by NAC. NAC pretreatment almost completely block Cuc B induced γH2AX expression and subsequent G2/M phase arrest suggesting that Cuc B induced DNA damage was ROS dependent. Furthermore, Cuc B induced DNA damage response pathways was also blocked by NAC pretreatment suggesting that ROS might be an early event before DNA damage. Collectively, these results suggested that Cuc B mediated DNA damage and subsequent G2/M phase arrest is due to Cuc B induced intracellular ROS production.

**Figure 7:**
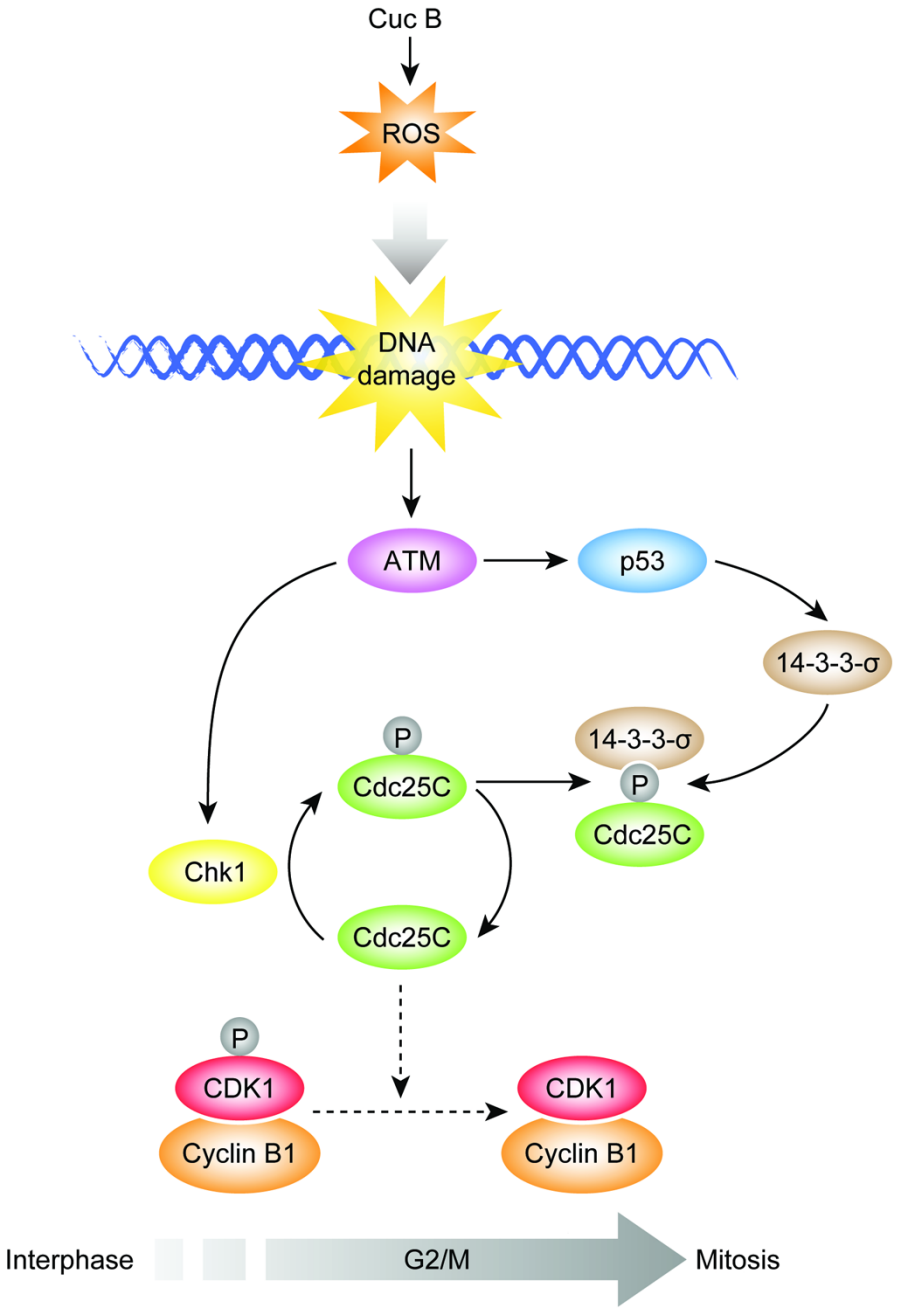
Schematic representation of the signaling pathways involved in the Cuc B induced G2/M checkpoint in A549 cells. Cuc B induced ROS formation, which lead to DNA damage. In response to DNA damage, ATM *via* autophosphorylation activated G2/M checkpoint. The downstream effectors of ATM, Chk1 and p53, were both activated, which resulted in Cdc25C phosphorylation and increased expression of 14-3-3-σ respectively. Phosphorylation of Cdc25C on Ser-216 by activated Chk1 facilitated the binding of 14-3-3-σ to Cdc25C, which prevents Cdk1 dephosphorylation, and keeps Cdk1-Cyclin B1 complex inactivated, causing G2/M phase arrest.

In summary, as depicted in Figure 7, present study dissected STAT3 independent pathways accounting for Cuc B induced G2/M phase arrest in A549 cells. Cuc B induced DNA damage mediated by increasing intracellular ROS formation, which in turn activated ATM-Chk1-Cdc25C-Cdk1 pathway joined by ATM-p53-14-3-3-σ branch, leading to the G2/M phase arrest. These data provided new insights into the anti-cancer mechanisms of Cuc B to regulate the G2/M phase arrest and thus shedding new insights into the molecular target identification for Cuc B and related compounds.

## Supporting Information

Figure S1 Effect of Cuc B on A549 proliferation. A549 cells were treated with different concentrations of Cuc B (0.94–60 µM) for 24 and 48 h and the cell viabilities was determined.
(TIF)

Figure S2 Cuc B induced DNA damage on MCF-7 breast cancer cells. MCF-7 cells were treated with 50 nM Cuc B for 3 h, the comet assay was performed. The protein expression of γH2AX was determined after different concentrations of Cuc B (50,100, 200 nM) treatment by Western blotting.
(TIF)
