# Supplementary figures and images for: Cucurbitacin B Induced ATM-Mediated DNA Damage Causes G2/M Cell Cycle Arrest in a ROS-Dependent Manner
Source: PLoS One. 2014 Feb 4;9(2):e88140. doi: 10.1371/journal.pone.0088140 (PMC3913755; doi:10.1371/journal.pone.0088140)

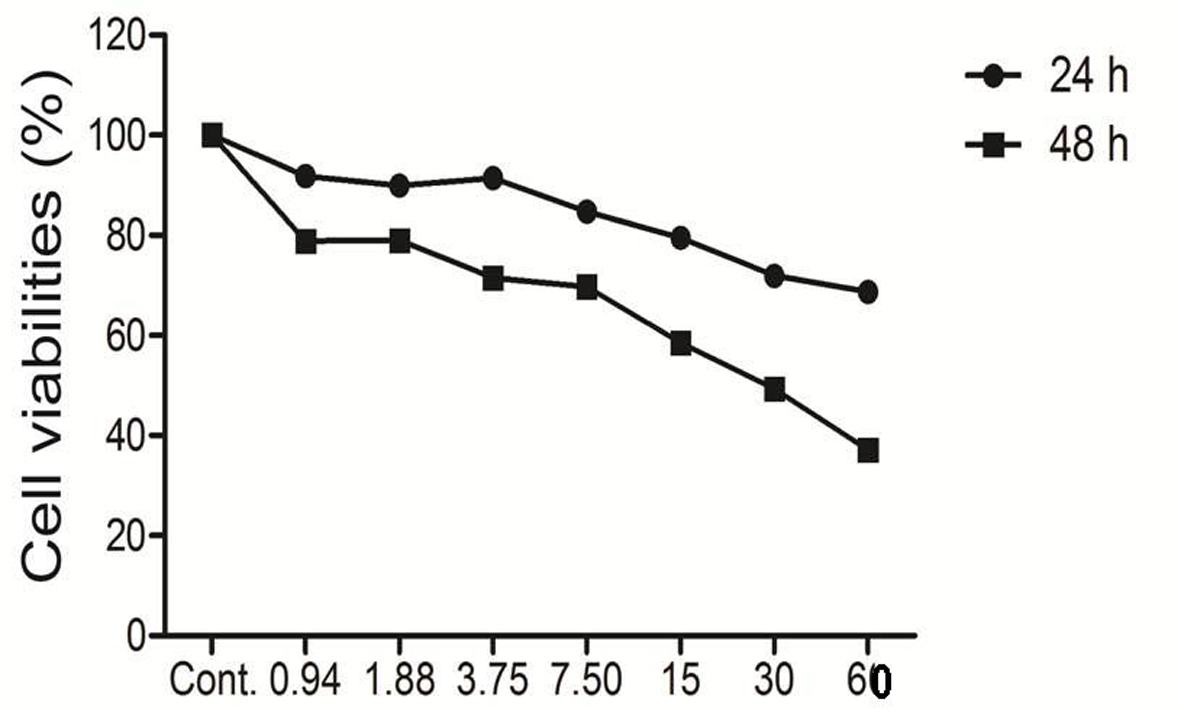

Supplement: Figure S1 — Effect of Cuc B on A549 proliferation. A549 cells were treated with different concentrations of Cuc B (0.94–60 µM) for 24 and 48 h and the cell viabilities was determined. (TIF) [file pone.0088140.s001.tif]

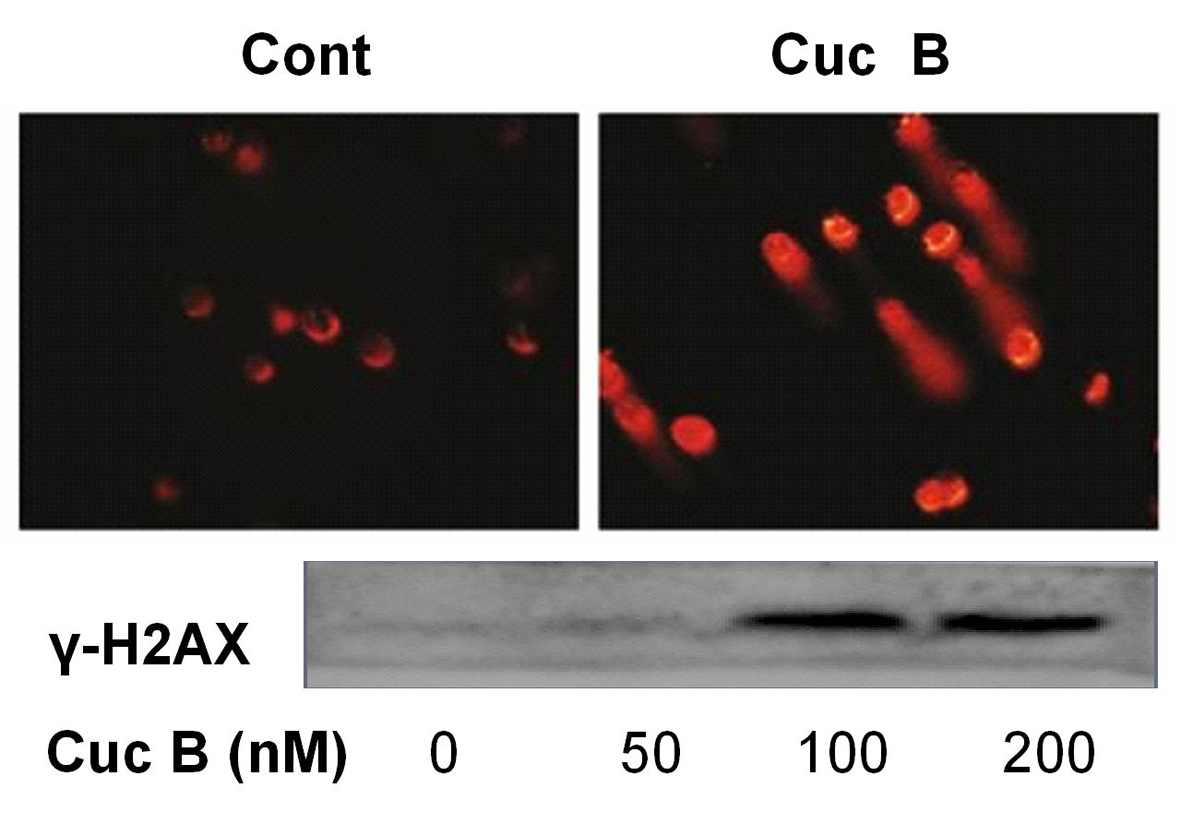

Supplement: Figure S2 — Cuc B induced DNA damage on MCF-7 breast cancer cells. MCF-7 cells were treated with 50 nM Cuc B for 3 h, the comet assay was performed. The protein expression of γH2AX was determined after different concentrations of Cuc B (50,100, 200 nM) treatment by Western blotting. (TIF) [file pone.0088140.s002.tif]
